# Acute Dermatotoxicity of Green-Synthesized Silver Nanoparticles (AgNPs) in Zebrafish Epidermis

**DOI:** 10.3390/toxics13070592

**Published:** 2025-07-15

**Authors:** Grace Emily Okuthe, Busiswa Siguba

**Affiliations:** Department of Biological and& Environmental Sciences, Walter Sisulu University, P/B X1, Mthatha 5117, South Africa; busiswasiguba@gmail.com

**Keywords:** *Danio rerio*, silver nanoparticles (AgNPs), zebrafish, epidermal toxicity, aquatic toxicity, mucosal homeostasis, ecotoxicity, mucus cell, mast cells

## Abstract

Silver nanoparticles (AgNPs), lauded for their unique antibacterial and physicochemical attributes, are proliferating across industrial sectors, raising concerns about their environmental fate, in aquatic systems. While “green” synthesis offers a sustainable production route with reduced chemical byproducts, the safety of these AgNPs for aquatic fauna remains uncertain due to nanoparticle-specific effects. Conversely, mast cells play crucial roles in fish immunity, orchestrating innate and adaptive immune responses by releasing diverse mediators and recognizing danger signals. Goblet cells are vital for mucosal immunity and engaging in immune surveillance, regulation, and microbiota interactions. The interplay between these two cell types is critical for maintaining mucosal homeostasis, is central to defending against fish diseases and is highly responsive to environmental cues. This study investigates the acute dermatotoxicity of environmentally relevant AgNP concentrations (0, 0.031, 0.250, and 5.000 μg/L) on zebrafish epidermis. A 96 h assay revealed a biphasic response: initial mucin hypersecretion at lower AgNP levels, suggesting an early stress response, followed by a concentration-dependent collapse of mucosal integrity at higher exposures, with mucus degradation and alarm cell depletion. A rapid and generalized increase in epidermal mucus production was observed across all AgNP exposure groups within two hours of exposure. Further mechanistic insights into AgNP-induced toxicity were revealed by concentration-dependent alterations in goblet cell dynamics. Lower AgNP concentrations initially led to an increase in both goblet cell number and size. However, at the highest concentration, this trend reversed, with a significant decrease in goblet cell numbers and size evident between 48 and 96 h post-exposure. The simultaneous presence of neutral and acidic mucins indicates a dynamic epidermal response suggesting a primary physical barrier function, with acidic mucins specifically upregulated early on to enhance mucus viscosity, trap AgNPs, and inhibit pathogen invasion, a clear defense mechanism. The subsequent reduction in mucin-producing cells at higher concentrations signifies a critical breakdown of this protective strategy, leaving the epidermis highly vulnerable to damage and secondary infections. These findings highlight the vulnerability of fish epidermal defenses to AgNP contamination, which can potentially compromise osmoregulation and increase susceptibility to threats. Further mechanistic research is crucial to understand AgNP-induced epithelial damage to guide sustainable nanotechnology.

## 1. Introduction

The rapid advancement of nanotechnology has led to the production of diverse nanoparticles (NPs) that exhibit unique physicochemical properties compared to their bulk counterparts. Nanomaterials (NMs), defined by at least one dimension below 100 nm [1,2,3,4], possess a significantly increased surface area to volume ratio. This characteristic enhances their reactivity [5,6,7], raising concerns about potential cellular-level tissue damage. Consequently, various NPs are now prevalent in a wide array of consumer products, including electronics [8,9], paints [10,11], dyes [12,13], and cosmetics [14,15,16,17], as well as biomedical applications [18,19,20]. The widespread integration of NMs into everyday items underscores the urgent need for a comprehensive assessment of their potential ecological consequences and the mechanisms by which they impact organisms and aquatic environments [21,22,23,24]. The escalating production and utilization of nanomaterials are likely to result in their introduction into freshwater ecosystems, posing potential ecological risks to aquatic organisms [21,22,23,24]. Notably, aquatic environments are particularly vulnerable, often serving as the ultimate sink for a substantial fraction of these nanomaterials [25,26].

Because of their potent antibacterial activity, silver nanoparticles (AgNPs) are among the most widely used NPs, appearing in preservatives and many consumer products [27,28,29,30]. The well-established antimicrobial action of silver ions (Ag+) involves the inhibition of bacterial cellular respiration and protein function through binding mechanisms [31,32,33]. Research indicates that AgNP toxicity is not uniform, but rather varies depending on particle size, stabilizing agents, the exposed organism, and exposure duration [34,35,36]. Furthermore, the nanoscale dimensions of AgNPs allow for potential deep organ penetration, raising concerns about disruptions to normal metabolic and physiological functions. Consistent research has documented the pathological effects of waterborne nanosilver on fish tissues and organs [37,38,39,40]. A key mechanism of AgNP toxicity in fish involves the inhibition of gill Na+ and K+-ATPase, leading to disrupted ion regulation and subsequent mortality [41,42,43]. This aquatic toxicity is often attributed to the release of Ag+ into the environment as waste, both intentional and unintentional. For instance, washing experiments demonstrated that clothing containing AgNPs can release substantial quantities of Ag+ [44]. However, a comprehensive understanding of the fate and behavior of these nanoparticles within diverse aquatic ecosystems remains limited.

The increasing demand for the responsible development of nanoparticles (NPs), necessitating a balanced and transparent evaluation of benefits and risks for specific applications, has driven interest in biogenic synthesis methods. These “green chemistry” approaches, utilizing organic reducing agents such as polysaccharides and biological microorganisms, offer a potentially simpler and more environmentally benign alternative to chemical methods for AgNP production [45,46].

The primary mechanism of AgNP toxicity, as noted, stems from the release of silver ions (Ag+) in aquatic environments. This ion release damages cell membranes, disrupts enzyme function, and causes oxidative stress in aquatic organisms, regardless of the synthesis method. Green coatings, notably, may not entirely prevent this release. Furthermore, the nanoparticles themselves (considering size, shape, and surface charge) can interact with biological systems in unique ways, including direct physical damage, cellular uptake, and interference with biological processes [47]. It is crucial to recognize that green synthesis does not eliminate the inherent nano-specific effects contributing to AgNP toxicity. While these production methods are environmentally benign, the resulting nanoparticles retain intrinsic properties like their minute size and high surface reactivity. These characteristics persist, enabling them to induce cellular stress and damage in biological systems. Furthermore, green-synthesized AgNP characteristics are highly variable, influenced significantly by the specific biological source, extraction methods, and reaction conditions. This lack of standardization makes robust safety generalizations and predictions of environmental behavior exceedingly difficult.

Once released into aquatic environments, AgNPs, regardless of their synthesis route, undergo complex transformations [47]. These dynamic processes include aggregation, dissolution, sulfidation, and interaction with natural organic matter, profoundly altering their size, surface charge, and stability. Critically, these transformations directly impact their toxicity and bioavailability to aquatic organisms, underscoring the urgent need for comprehensive environmental risk assessment that extends beyond initial synthesis characteristics [47].

The fish immune system, unlike that of other animals, consists of innate and adaptive components [48]. The innate system, comprising epithelial/mucosal barriers, humoral parameters, and immune cells, provides the initial response to environmental challenges and is vital for disease resistance. The fish gills, skin, and gut serve as these crucial first-line mucosal barriers, allowing environmental interaction while maintaining homeostasis [48].

Mucosal barriers contain immune cells, effector molecules [49], and a biologically active mucus layer. In teleost fish, mast cells, alongside mucus cells, contribute to innate immunity. Beyond defense, mucosal structures have other physiological roles: skin in osmotic balance and sensory reception, gills in osmotic, ionic, and acid-base regulation and nitrogenous waste excretion, and the gut in nutrient uptake and catabolism [50].

Often located in the superficial dermis near vessels, nerves, and muscles [51], mast cells are pivotal in normal and pathological immune responses. Situated at the interface of tissues and the external environment, these multifunctional cells are increasingly recognized for their key role in host defense within vascularized tissues, participating in both innate and adaptive immunity. As an initial defense, they secrete antimicrobial peptides (AMPs) and migrate to infection sites, releasing bioactive compounds that initiate the first line of defense. Their influence on both immune arms and involvement in wound healing and tissue remodeling underscore the importance of understanding their biology in non-mammalian vertebrates. Goblet cells, specialized epithelial cells, primarily secrete mucin, forming a protective mucus layer on mucosal surfaces. They contain glycoproteins, lectins, lysosomes, and C-reactive proteins, including antimicrobial peptides and immunoglobulins [52].

Environmental factors in aquatic habitats significantly impact the health and function of both mast and goblet cells in fish. For instance, stress stemming from suboptimal conditions can worsen mast cell-mediated inflammatory diseases [53]. Fish mucosal surfaces, including goblet cells and their secreted mucus, are highly responsive to even subtle environmental changes, such as fluctuations in pH and carbon dioxide levels [50]. Critically, various environmental stressors can broadly compromise epithelial barrier integrity, potentially leading to increased permeability and dysregulation of the fish’s immune system.

Conversely, fish bacterial communities, interacting closely with goblet cell-mediated mucosal immunity, are also sensitive to water’s physicochemical parameters [54]. Water temperature and oxygen levels, critical abiotic factors, broadly affect fish physiology, indirectly impacting mast and goblet cells by altering metabolic rates and immune competence [55]. Ingesting pollutants like microplastics may also affect a fish’s immune function. Environmental bacterial components, such as *Staphylococcus aureus* biofilm-secreted factors, can cause mucosal damage and affect mast cell infiltration and goblet cell hyperplasia [56].

Water temperature is another fundamental environmental factor that significantly impacts fish physiology, including reproduction, and profoundly influences the overall health and functionality of mast and goblet cells [57]. Elevated water temperatures, acting as a direct stressor, can alter the intricate balance of the intestinal microbiota, subsequently affecting goblet cell-mediated mucosal immunity [58]. This dysbiosis can reduce the production of protective mucins and antimicrobial peptides, diminishing the gut’s barrier function.

Furthermore, direct exposure to a wide array of pollutants and xenobiotics can specifically disrupt epithelial barriers, leading to increased permeability and intensifying systemic immune dysregulation [59]. This includes not only well-known contaminants but also emerging pollutants (e.g., microplastics, pharmaceuticals, personal care products) which have been shown to exert diverse toxic effects on fish mucosal organs, altering microbial composition, inducing oxidative stress, and impairing overall mucosal function and immune response [57,60]. These disruptions can manifest as reduced mucus integrity, altered immune cell profiles, and heightened susceptibility to pathogens. Even dietary factors in aquaculture play a crucial role, influencing not only intestinal morphology but also the expression of inflammatory cytokines, underscoring the diet’s profound impact on mucosal immunity [61]. For instance, nutrient deficiencies or imbalanced feeds can compromise the delicate balance of the gut microbiome and the integrity of the intestinal barrier. Collectively, these findings highlight the significant, multifaceted, and often interconnected ways in which various environmental factors, ranging from physicochemical parameters to chemical contaminants and nutritional inputs, critically influence the health, integrity, and immune effectiveness of mast and goblet cells in fish.

As stated above, the epidermal mucus layer serves as a crucial biological and mechanical barrier in fish, representing the primary interface with the aquatic environment. This complex secretion is rich in diverse protective components, including lectins and carbohydrate-binding proteins, which facilitate agglutination and neutralization of pathogens, thereby contributing significantly to non-specific innate immunity independent of enzymatic or antibody-mediated mechanisms. Furthermore, fish external body mucus contains a wide array of resistance factors, such as immunoglobulins, C-reactive protein, complement proteins, lysozyme, and hemolysin [62,63,64]. These constituents collectively provide a robust, multifaceted defense against infections and external stressors by trapping foreign particles, exhibiting direct antimicrobial activity, and modulating host immune responses.

Given this critical barrier function, the interaction of nanoparticles (NPs) with biological systems, particularly the immune system and surface epithelia, is paramount regardless of the exposure route. Research specifically demonstrates that the fish epidermis, including its essential mucus layer, actively responds to waterborne NP exposure, underscoring its role as a dynamic primary interface for interaction [65,66,67,68]. Nanoparticles can elicit complex immunomodulatory effects; even at low concentrations, some may trigger specific immune responses, indicating either immunostimulatory or immunosuppressive potential [69,70,71,72]. Such alterations can significantly compromise an organism’s overall physiological homeostasis and increase its susceptibility to disease.

For instance, numerous studies on zinc oxide nanoparticles (ZnO-NPs) administered through dietary routes have consistently documented concerning toxicological outcomes. These include direct cytotoxicity, the induction of significant oxidative stress, perturbations in essential blood biochemical parameters, and overt tissue damage [73,74,75]. Critically, similar detrimental effects have been observed even at lower doses of ZnO-NPs, highlighting their potent biological activity. Furthermore, specific investigations have reported that exposure to ZnO-NPs demonstrably compromises the antioxidant defense system in species like Nile tilapia [76]. These findings collectively reinforce a broader understanding that NP-induced toxicity is predominantly mediated through the generation of oxidative stress [77,78]. This occurs either by overwhelming intrinsic cellular antioxidant systems or by directly promoting the excessive production of reactive oxygen species (ROS) [78,79], ultimately leading to widespread cellular damage, impaired physiological function, and a compromised immune response.

Despite the growing adoption of green synthesis methods for nanoparticle (NP) production, a critical disparity exists in comprehensive ecotoxicity research. Studies directly comparing the environmental effects of these “green” NPs with those of conventionally synthesized NPs across diverse aquatic organisms and trophic levels remain remarkably limited. A particularly significant knowledge gap persists regarding the impact of green-synthesized nanoparticles on sensitive fish epidermal tissues. This oversight is critical because the epidermis serves as the primary and most expansive interface between fish and their aquatic environment, mediating initial contact, absorption, and protective responses to external stressors. Consequently, the true environmental footprint and long-term ecological safety of these purportedly “green” materials cannot be adequately assessed without targeted toxicological investigation.

This study directly addresses this critical need by investigating the in vivo histopathological effects of green-synthesized AgNPs produced using environmentally friendly, natural product-based reducing and capping agents on the epidermis of *D. rerio*. Such targeted research is indispensable not only for elucidating the specific mechanisms of green AgNP-induced toxicity but also for informing effective environmental management, developing robust risk assessment strategies, and ultimately ensuring the sustainable and responsible application of nanotechnology. This work provides foundational data essential for regulatory bodies to make informed decisions about the safe deployment and disposal of these increasingly prevalent materials.

## 2. Materials and Methods

### 2.1. Fish Maintenance and Experimental Set-Up

Adult zebrafish (*Danio rerio*) of an undefined commercial strain were acquired from a local supplier (Northland Pets, Durban, South Africa). Upon arrival, fish underwent an acclimatization period of at least two weeks in 40 L stock aquaria. All tanks were supplied with dechlorinated tap water and equipped with aerators to ensure adequate oxygen dissolution [40].

The zebrafish were maintained under controlled conditions with a 14:10 h light/dark photoperiod at a constant temperature of 28.4 °C. Breeding was conducted within the stock tanks following the established protocols described by [80]. Mature fish were fed twice daily with freshwater Aquarium Flakefood (TetraMin, Tetra GmbH, Hamburg, Germany). To maintain water quality, uneaten food was siphoned from the tanks prior to each feeding [40].

### 2.2. Chemicals

The aqueous AgNPs used in this in vivo study on adult zebrafish were synthesized and characterized by [81], with a TEM-determined average diameter of 3.76 ± 1.00 nm. Silver nitrate (AgNO_3_), gelatin (99%, Merck, Darmstadt, Germany), and maltose (Fluka PG, CH-9470; Buchs, Switzerland) were used as the silver precursor, capping agent, and reducing agent, respectively. All reagents were of analytical-grade purity and used as received without further purification. To prevent potential photochemical reactions, all experimental procedures involving these chemicals were conducted in the dark.

### 2.3. Green Synthesis and Characterization of Maltose-Reduced Silver Nanoparticles (Ag-NPs)

The aqueous AgNPs used in this in vivo study on adult zebrafish were synthesized and characterized by [81], with a TEM-determined average diameter of 3.76 ± 1.00 nm. Briefly, 1.0 g of gelatin was accurately weighed and added to 95 mL of distilled water in a clean glass flask. The mixture was continuously stirred on a hot plate and heated to 40 °C until the gelatin was completely dissolved, yielding a clear solution. Subsequently, 5 mL of a 1 M silver nitrate (AgNO_3_) solution was slowly added to the gelatin solution under continuous magnetic stirring, forming an Ag+/gelatin precursor solution. Following this, 10 mL of a 2 M maltose solution was introduced into the mixture, also under continuous stirring. The reaction solution was maintained at a constant temperature of 40 °C and allowed to react for 24 h to ensure complete reduction of silver ions and nanoparticle formation. All steps were performed with careful attention to cleanliness to prevent contamination [81]. The synthesized nanoparticles were characterized to determine their optical properties and morphology. Ultraviolet-visible (UV-Vis) absorption spectra were recorded using a Perkin Elmer Lambda 25 UV-Vis spectrophotometer (PerkinElmer, Waltham, MA, USA). Spectra were collected in the wavelength range of 200–1100 nm, with samples placed in standard quartz cuvettes (1.0 cm path length) [81].

For detailed morphological analysis, including particle size, shape, and distribution, a JEOL 2100 transmission electron microscope (TEM) (JEOL Ltd., Tokyo, Japan) operating at an accelerating voltage of 200 KV was utilized. High-resolution transmission electron microscopy (HRTEM) measurements were also performed to examine the crystalline structure and lattice spacing of the individual nanoparticles. Prior to TEM and HRTEM analysis, a drop of nanoparticle dispersion was placed on a carbon-coated copper grid and allowed to dry at room temperature [81].

### 2.4. Toxicity Testing of AgNPs

Adult male and female zebrafish (standard length 28.1 ± 0.2 mm) were exposed to nominal AgNP concentrations of 0.031 μg/L, 0.257 μg/L, and 5.000 μg/L, along with a control group. The concentrations in this study were based on the previously published 96 h LC50 value for AgNPs in Japanese medaka (*Oryzias latipes*), which was 0.9 mg/L [82]. The exposure lasted 96 h under a 24 h static renewal system. The control group received only aquarium water. Exposure treatments were conducted in 20 L aerated glass tanks, into which the eAg-NP solutions were introduced. Fifteen randomly selected adult fish were exposed to each concentration and control (five fish per replicate). Fish were not fed during the exposure period to prevent nanoparticles from adhering to food particles. Debris was siphoned daily from each experimental unit. At 24, 48, and 96 h of exposure, five fish from each concentration and control group were sampled for histopathological analysis [40].

### 2.5. Preparation of Engineered Silver Nanoparticle Suspensions (eAg-NPs)

Aqueous suspensions of engineered silver nanoparticles (eAg-NPs) were prepared by directly adding the desired doses into the tank water to achieve the target concentrations using the following equation:

M_1_V_1_ = M_2_V_2_

where M_1_ represents the initial concentration, V_1_ represents the initial volume of your solution, M_2_ represents the final concentration, and V_2_ represents the final volume of the diluted solution. No further dilutions were performed.

### 2.6. Sample Fixation and Tissue Processing

Fish from each concentration were humanely sacrificed by anesthetizing them with tricaine methanosulfonate (MS222^®^, Sigma-Aldrich, St. Louis, MO, USA). A stock solution of 4.2 mL tricaine in 100 mL tank water was used, as described by [40]. The total length of each fish was measured, and the trunk and head regions were excised before fixation. These regions were fixed in Davidson’s solution overnight at room temperature, briefly rinsed with tap water, and then transferred directly to 70% ethanol.

After fixation, the trunk and head regions were processed for embedding. All fixed tissues were dehydrated through an ethanol series (80% to 100%) and cleared in methyl salicylate (Sigma-Aldrich, St. Louis, MO, USA) before being embedded in Paraplast^®^ wax (Merck, Darmstadt, Germany).

Paraplast blocks were trimmed to the tissue surface, and sections were cut at a thickness of 3–5 μm. Tissue sections were floated in a water bath at 37 °C and placed on glass slides pre-treated with 2% 3-aminopropyl triethoxysilane (Sigma-Aldrich, St. Louis, MO, USA) in acetone. Sections were then dewaxed in xylene, hydrated in an ethanol series [REF], and stained with Mayer’s modified hematoxylin and counterstained with eosin [40]. Images of stained material were acquired using a Leica DM 750 fluorescent microscope (Leica Microsystems, Wetzlar, Germany), equipped with a DFX 310 FX digital camera, and analyzed using Leica LAS imaging software version 4.5.

### 2.7. Tissue Histochemistry

Alcian blue (AB pH 2.5) stain was used as an alternative stain for skin tissue sections to determine the level of staining of carboxylated acid mucopolysaccharides and sulfated carboxylated glycoproteins [83]. Tissue sections were deparaffinized and hydrated to distilled water. The tissue was then stained with Alcian blue for 30 min, rinsed in running tap and distilled water, and then counterstained in nuclear fast red solution. Finally, tissues were rinsed with changes in ethanol and cleared in xylene.

Epidermal cell architecture and morphology were quantitatively assessed at 24, 48, and 96 h post-exposure. From each experimental group, n = 4 fish were randomly selected, and n = 15 representative cells were measured per fish (totaling 60 cells per time point per group) from randomly chosen histological sections. Cell size measurements were performed using the same software and microscope mentioned above.

To accurately capture the dimensions of both round and oval cells, two orthogonal measurements were taken for each cell: the longest axis and a perpendicular measurement across its shortest dimension [84]. This dual measurement strategy provided an indicator of overall cell size, mitigating inaccuracies that could arise from single-axis measurements on irregularly shaped cells. These measurements were used to derive an average cell diameter and to calculate an approximate cell area, providing a more comprehensive characterization of cellular changes. Furthermore, to evaluate histochemical alterations within the epidermis, adjacent sections were stained with Periodic Acid-Schiff (PAS) and Alcian Blue (AB pH 2.5). These staining procedures enabled the reasonable visualization and assessment of carbohydrate content and mucopolysaccharide distribution.

The severity of the impact of nanoparticles on epidermal tissues was determined through a subjective scoring system, employing arbitrary scores to quantify observed pathological changes.

## 3. Results

Across all experimental groups, no fish mortality was recorded.

### 3.1. Histopathology of AgNPs on Zebrafish Skin

**Figure 1:**
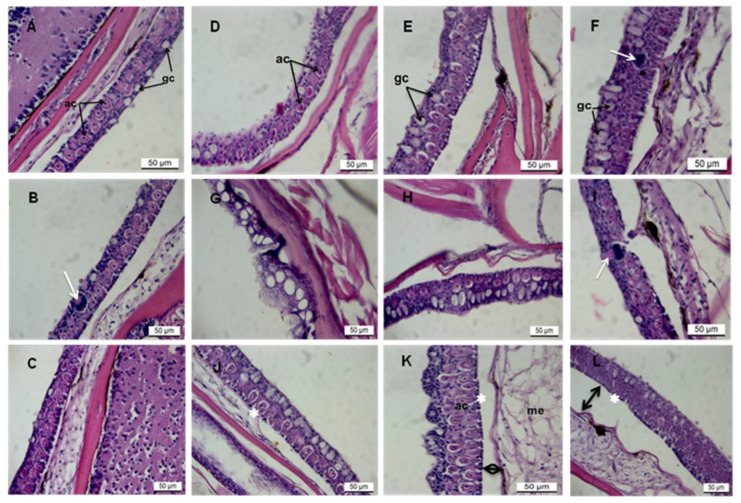
Longitudinal sections of zebrafish skin from treatment with various concentrations of AgNPs. H& E stain. Scale bar = 50 µm. (**A**–**C**) shows control images of fish skin from the head and trunk regions of the fish body. (**D**–**F**) shows fish skin following exposure to 0.031 μg/L of AgNP at 24, 48, and 96 h, respectively. (**G**–**I**) shows fish skin following exposure to 0.250 μg/L of AgNP at 24, 48, and 96 h, respectively. (**J**–**L**) shows fish skin following exposure to 5.000 μg/L of AgNP at 24, 48, and 96 h, respectively. White arrows in Figure 1 show taste bud cells, and asterisks (*) in Figure 2. (**J**–**L**) show lacerated epithelium; double-sided arrows in Figure 2 (**L**) indicate shrunk cytoplasm. Abbreviations: ac, alarm cells; gc, goblet cells; mu, mucous.

The zebrafish epidermis consisted of a stratified squamous epithelium with interspersed mucous cells overlying the dermis (Figure 1A,B). The outermost layer’s epidermis comprised squamous, cuboidal, and secretory cells with well-rounded nuclei (Figure 1A–C). Secretory cells include alarm and mucous goblet cells located on the epidermal surface. Control fish at 24 h showed fewer normal-sized goblet cells (11.17 ± 3.148) with an average size of 12.71 ± 2.084 μm and regularly shaped alarm cells (Figure 1A–C). Goblet cells appeared as unstained (white), often elliptical circles, while alarm cells were spherical with eosinophilic cytoplasm and conspicuous central nuclei (Figure 1A–C).

The trunk region consistently displayed a higher density of epidermal cells than the trunk and tail. Pathological lesions were evident in all exposed groups, most notably at 5.000 µg/L at 96 h (Figure 1D–I). AgNP exposure also altered alarm cell shape, leading to vacuolated, irregular elliptical forms with pyknotic nuclei and intercellular spaces due to cytoplasmic shrinkage (Figure 1J–L and Table 1 and Table 2). Hyperplasia and hypertrophy of goblet cells occurred at 5.000 µg/L^−1^ at 96 h. Goblet cell numbers increased significantly at the lowest concentration (Figure 1F) up to 48 h and at the median concentration (Figure 1G,H), followed by a marked reduction in number and size (Figure 1I–L). AgNP exposure resulted in a decrease in both goblet cell number (11.28 ± 1.074) and size (6.403 ± 1.583 µm) and also impacted alarm cell morphology (Figure 1D–I; Table 1).

### 3.2. Histochemical Analysis of the Epidermis PAS/AB pH 2.5 Stains

**Figure 2:**
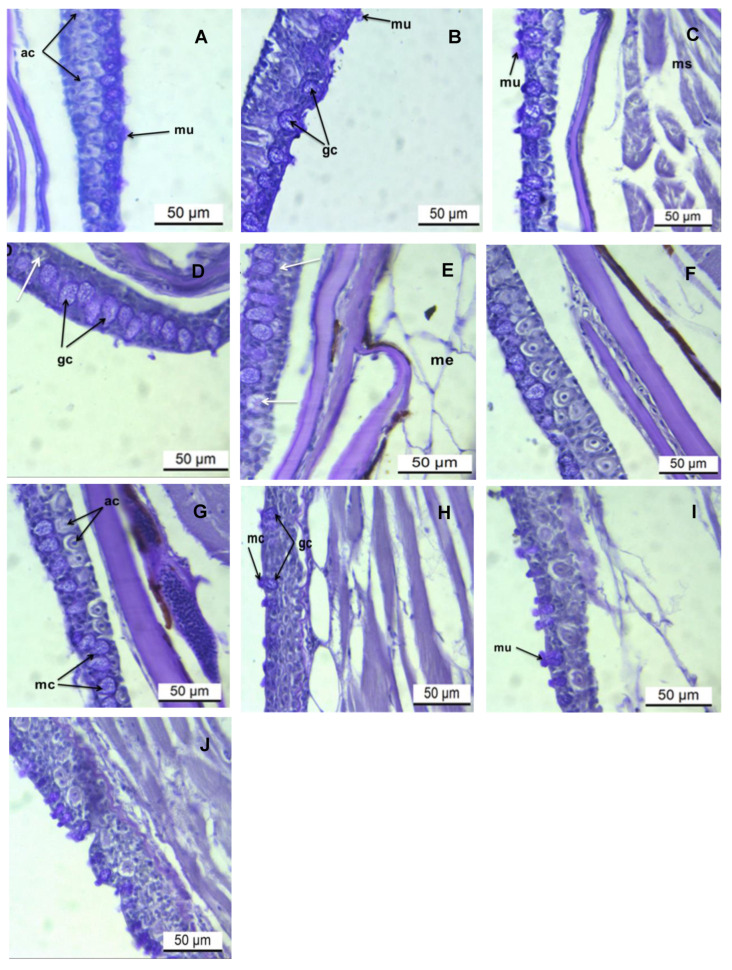
Longitudinal sections of zebrafish skin from treatment with various concentrations of AgNPs. PAS stain. Scale bar = 50 µm. (**A**) shows a control slide. (**B**–**D**) shows fish skin following exposure to 0.031 μg/L of AgNP at 24, 48, and 96 h, respectively. (**E**–**G**) shows fish skin following exposure to 0.031 μg/L of AgNP at 24, 48, and 96 h, respectively. (**H**–**J**) shows fish skin following exposure to 0.031 μg/L of AgNP at 24, 48, and 96 h, respectively. Abbreviations: ac, alarm cells; gc, goblet cells; mu, mucous; white arrows indicate vacuolated irregularly shaped alarm cells.

**Figure 3:**
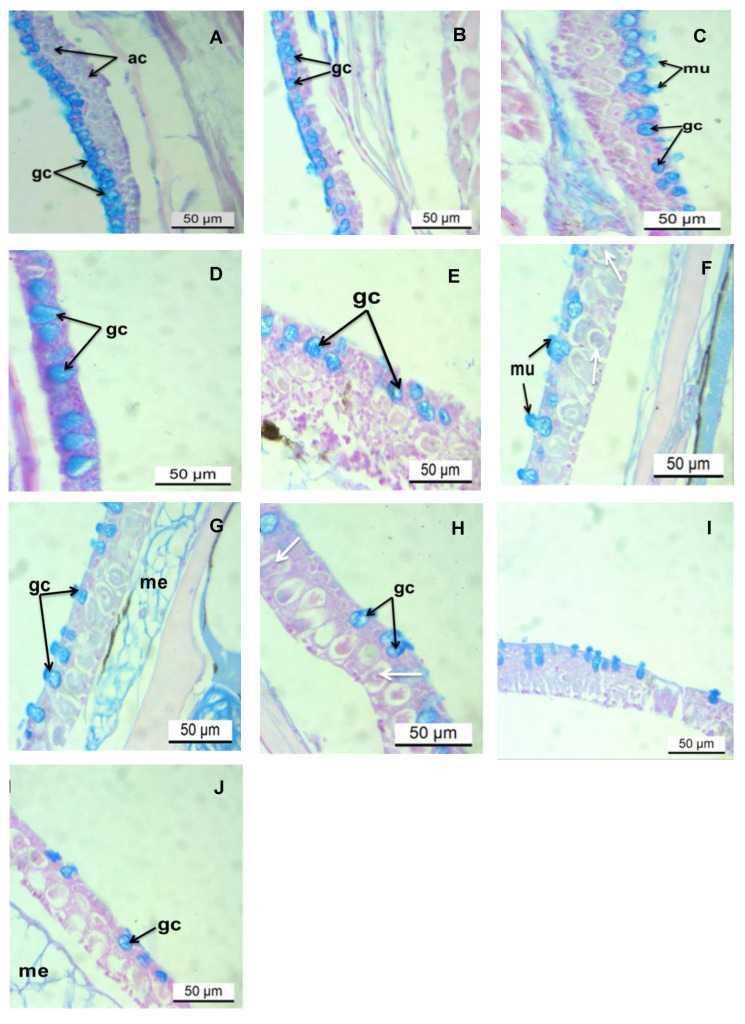
Longitudinal sections of zebrafish skin from treatment with various concentrations of AgNPs. Alcian blue (AB pH 2.5) stain. Scale bar = 50 µm. (**A**) shows a control slide. (**B**–**D**) shows sections exposed to 0.031 μg/L, 24, 48, and 96 h, respectively. (**E**–**G**) shows sections exposed to 0.250 μg/L, 24, 48, and 96 h, respectively. (**H**–**J**) show sections exposed to 5.000 μg/L, 24, 48, and 96 h, respectively. Abbreviations: ac, alarm cells; gc, goblet cells; mu, mucous; me, mesenchyme; the white arrows indicates irregularly shaped alarm cells.

Histochemical analysis detected the prevalence of mucous cells containing both acid (AB pH 2.5) and neutral glycoconjugates (PAS) stains. Initial exposure to AgNP demonstrated an intense PAS-positive reaction in goblet cells at the lowest concentration (0.031 μg/L) (Figure 2B–D). On increasing the level of exposure to 0.250 μg/L of AgNP, the intensity of the PAS reaction was moderate (Figure 2E–G). At 5.000 μg/L of AgNP, the signal of the PAS reaction was intense (deep purple), comparable to that seen at the lowest concentration (Figure 2H–J; Table 2). However, the cytoplasm of club cells demonstrated weak PAS staining. Staining with AB pH 2.5, goblet cells showed intense cyan staining at the lowest (24 h–96 h) and medial (24 h; Figure 3B–E,F) and moderate staining at higher concentrations (Figure 3E–I). The cytoplasm of the alarm cell was AB pH 2.5 negative.

## 4. Discussion

This study offers an original contribution by examining the potential toxicity of “green” synthesized silver nanoparticles (AgNPs) on the structural integrity of adult zebrafish epidermis. While the research focused on epidermal tissue toxicity in fish, the AgNP synthesis protocol was designed for environmental relevance. It utilized biocompatible, biodegradable, and renewable materials with water as the solvent, deliberately avoiding potentially toxic accelerators. This approach minimized confounding environmental toxicities, enabling a more ecologically pertinent assessment [81]. Characterization of the resulting AgNPs indicated high colloidal stability and uniform dispersion, likely due to the effective steric stabilization provided by the amine pendant groups on the gelatin backbone, preventing aggregation [81].

While a substantial body of the literature has investigated the impact of AgNPs on various internal organs in fish [21,22,23,24], direct comparisons with the present findings are often limited by variations in NP synthesis methodologies, physicochemical properties (including size, shape, and surface charge), and the specific target organs examined. Notably, the AgNP concentrations employed in this study (0.031, 0.250, and 5.000 μg/L) were considerably lower than the reported 96 h LC50 values (24.5 µg/L) from other zebrafish studies utilizing conventionally synthesized AgNPs [85]. Considering the well-established principle of size-dependent nanoparticle toxicity, where smaller particles generally exhibit enhanced bioavailability and thus greater toxicity [86], the observed histopathological effects on zebrafish epidermis at these relatively low exposure concentrations underscore the potential for even “green” synthesized AgNPs to exert significant biological impacts. Transmission Electron Microscopy confirmed the small size (3.76 ± 1.00 nm) and good dispersity of the synthesized AgNPs, likely enhancing their interaction with the epidermal tissue [87]. The observed histological and histochemical lesions are strongly suggestive of a direct toxic effect of the AgNPs, rather than the gelatin capping agent or maltose reducing agent, given their common presence in dietary contexts without reported acute histopathological effects at comparable concentrations. However, future studies could incorporate control groups exposed solely to the capping and reducing agents to definitively rule out any synergistic or independent effects.

### 4.1. Rapid Mucus Production as a Fish Stress Response to AgNP Exposure

The rapid increase in mucus production on the skin surface within the initial two hours of AgNP exposure across all experimental groups likely represents a conserved, non-specific stress response aimed at mitigating the initial exposure. Fish skin mucus, acting as the primary interface with the aquatic environment, performs critical functions in chemical communication, osmoregulation, physical protection, and defense against environmental toxins, heavy metals, and pathogens [48,50,52,59,88]. The observed rapid upregulation of mucus secretion can be interpreted as a physiological attempt to create a protective barrier, potentially trapping, immobilizing, or diluting the AgNPs, analogous to acute stress responses documented in mammals exposed to noxious stimuli [89].

Fish skin mucus acts as the primary interface between the internal physiological environment and the external aquatic milieu. Its multifaceted role encompasses chemical communication, osmoregulation, physical protection, and crucially, defense against environmental toxins, heavy metals, and pathogens. Composed primarily of water, mucopolysaccharides, mucoproteins, and various bioactive molecules (e.g., antimicrobial peptides, immunoglobulins, enzymes), mucus forms a dynamic physical and biochemical barrier [90,91].

The increased secretion of mucus creates a thicker, viscoelastic layer that physically impedes the direct contact of nanoparticles and dissolved ions with the delicate epithelial cells of the skin. This increased physical barrier reduces the surface area available for uptake and subsequent internalization of the xenobiotic. Furthermore, mucus contains numerous negatively charged glycoproteins and other organic molecules that can bind to positively charged metal ions (such as Ag+ released from AgNPs) and even interact with the surface of nanoparticles themselves through electrostatic, hydrophobic, or hydrogen bonding [92]. This binding capacity can effectively sequester and immobilize metals, reducing their bioavailability and preventing their absorption into the bloodstream. Once bound within the mucus, the contaminants can be sloughed off and continuously replenished, serving as a dynamic, self-renewing excretory pathway for foreign substances [93,94]. This active turnover helps clear the immediate epithelial surface of irritants.

Rapid and excessive mucus production (hyperplasia of goblet cells and increased secretion) is a classic histopathological indicator of acute stress and exposure to toxicants in fish [95,96]. The metabolic cost of producing and secreting large volumes of mucus can be substantial, potentially diverting energy from other vital physiological processes if exposure to AgNPs is prolonged or severe. This rapid, immediate response underscores that even AgNPs synthesized via purportedly benign “green” routes can elicit swift physiological alterations in aquatic organisms. This finding highlights a potential ecological risk associated with their increasing environmental prevalence in consumer products and industrial effluents. While this rapid surge in mucus production represents a crucial initial defense, demonstrating a fish’s inherent adaptability to environmental challenges, a comprehensive assessment of AgNP ecotoxicology requires understanding its limitations, the metabolic burden it imposes, and the ultimate environmental fate of the contaminated mucus. Further investigation into the specific mechanisms of AgNP-mucus interaction and the long-term consequences for fish health is therefore essential.

### 4.2. Goblet Cell Dynamics and Epidermal Integrity in Response to AgNP Exposure

The observed alterations in goblet cell dynamics following silver nanoparticle (AgNP) exposure provide crucial mechanistic insights into AgNP-induced toxicity in the fish epidermis. Specifically, an initial increase in goblet cell number and size at lower AgNP concentrations (0.031 and 0.250 µg/L) gives way to a significant decrease at the highest concentration (5.000 µg/L) between 48 and 96 h. This biphasic response illustrates the complex interplay between the organism’s adaptive defenses and the escalating cytotoxic effects of the stressor.

Initially, the heightened proliferation and hypertrophy of goblet cells at lower AgNP concentrations represent an adaptive, compensatory stress response. Goblet cells, specialized epithelial cells, are responsible for synthesizing and secreting mucus, the primary protective barrier of the epidermis [94]. This cellular augmentation indicates the fish’s attempt to enhance its mucus layer, thereby physically impeding AgNP uptake and potentially sequestering dissolved silver ions (Ag+) released from the nanoparticles. This initial phase highlights the organism’s capacity for physiological acclimation or resistance to mild AgNP exposure.

However, the subsequent decline in goblet cell number and size at the highest AgNP concentration and prolonged exposure signifies a shift from adaptation to cellular damage and pathological impairment. This reduction suggests that the toxic burden at higher concentrations overwhelms the reparative and adaptive capacities of the epidermal tissue. This can result from direct cytotoxicity, where high concentrations of AgNPs or released Ag+ ions induce necrosis or apoptosis [68], thereby reducing viable goblet cells. Alternatively, chronic exposure might inhibit new goblet cell proliferation or lead to exhaustion of their secretory capacity under sustained demand. Furthermore, severe AgNP exposure can induce inflammation and widespread epidermal tissue damage [96], disrupting normal cellular architecture and impairing goblet cell function.

The subsequent significant decrease in goblet cell number and size at the highest AgNP concentration (5.000 µg/L) and prolonged exposure (48 to 96 h) signals a shift from an adaptive response to cellular damage and pathological impairment. This decline suggests that the toxic burden at higher concentrations overwhelms the reparative and adaptive capacities of the epidermal tissue.

The initial increase likely represents a compensatory cellular response aimed at enhancing mucus secretion and protecting the epidermis from the nanoparticle challenge, consistent with cellular adaptation mechanisms observed in response to various [48,50,52,59,88]. However, the subsequent decline in both the number and size of goblet cells at the highest concentration suggests a failure of this protective mechanism, potentially due to cellular damage, exhaustion of secretory capacity, or disruption of cellular differentiation and signaling pathways. The continued degradation of both the mucus goblet and alarm cells over time at the highest concentration strongly indicates that the AgNPs overwhelmed the skin’s defense capabilities, leading to progressive tissue damage.

The observation that increased mucus production did not ultimately prevent epidermal lesions suggests several possibilities. It could indicate efficient penetration of the mucus layer by the small AgNPs, or that the mucus itself, through complex interactions with the nanoparticles, might have facilitated their transport and distribution within the epidermal tissue, potentially exacerbating cytotoxic effects. Further investigation into the specific interactions between synthesized AgNPs and the mucus matrix is a valuable avenue for future research.

The epidermal lesions observed, including cellular swelling, epithelial lifting, and loss of cellular integrity, exhibit morphological similarities to those reported in fish exposed to other heavy metals [97,98,99] and silver and copper nanoparticles synthesized via conventional methods [67,100,101]. This similarity supports the hypothesis that AgNPs, irrespective of their synthesis route, can induce cellular stress and damage in fish skin. Additionally, the varying degrees of PAS and AB pH 2.5 positivity in the mucus cells of exposed fish indicate significant alterations in the biochemical composition of secreted mucins. The presence of both neutral and acidic mucins suggests a complex and dynamic response aimed at providing both physical barrier function and potentially interacting with AgNPs through mechanisms like chelation or aggregation. The initial upregulation of acidic mucins, known to enhance mucus viscosity and aid in trapping particulate matter and inhibiting pathogen invasion, likely represents a specific defense mechanism against the nanoparticles. However, the subsequent reduction in these mucin-producing cells at higher concentrations signifies a breakdown of this crucial protective strategy, potentially rendering the epidermis more vulnerable to further damage and secondary infections. Future studies employing advanced spectroscopic techniques could provide a more detailed biochemical characterization of the altered mucus composition.

Given the diverse and crucial roles of epidermal mucous and mast cells in vital physiological processes such as waste excretion, respiration, ion and osmotic regulation, disease resistance, and communication, their observed degradation following AgNP exposure has significant implications for fish health, fitness, and ultimately, survival within aquatic ecosystems. Recent research highlighting the potential of fish skin mucus as a non-invasive biomarker for assessing stress and welfare [102,103,104,105,106] further emphasizes the importance of understanding the impact of emerging contaminants like AgNPs on this readily accessible biological matrix. The findings of this study strongly suggest that alterations in epidermal morphology and mucus composition can serve as sensitive early indicators of AgNP-induced stress in fish populations, even at environmentally relevant concentrations of “green” synthesized nanomaterials. Future research should prioritize elucidating the long-term chronic effects of these AgNPs on fish epidermis and other organ systems, as well as investigating their potential for bioaccumulation and trophic transfer within aquatic food webs.

## 5. Conclusions and Future Perspectives

This study provides novel and significant evidence that AgNPs synthesized via environmentally benign “green” methods induce cellular stress and distinct histopathological alterations in the adult zebrafish epidermis. These findings underscore the vulnerability of fish skin as a primary interface for nanoparticle uptake from aquatic environments, with demonstrable ecological ramifications. Our results emphasize the urgent necessity for comprehensive and standardized toxicity assessments of diverse nanomaterials across a broad range of aquatic species and tissues, irrespective of their synthesis methodology. This is critical given the demonstrated potential of these “green” synthesized materials to disrupt fundamental physiological processes, such as osmoregulation and immune defense, and ultimately compromise the ecological fitness and survival of aquatic organisms. The observed epidermal lesions and altered mucus dynamics highlight a clear breakdown of the integumentary barrier, a crucial defense mechanism against environmental stressors.

Future research must prioritize long-term, chronic exposure studies employing environmentally relevant low-dose concentrations and complex environmental matrices to elucidate cumulative toxic effects and potential synergistic or antagonistic interactions with co-occurring contaminants. The development and implementation of standardized testing protocols are crucial for enabling robust inter-study comparisons and facilitating effective regulatory frameworks for nanomaterial use and disposal.

Mechanistic investigations at molecular and cellular levels, including transcriptomic and proteomic analyses, alongside comparative assessments with conventionally synthesized AgNPs of similar physicochemical characteristics, are essential to fully elucidate the pathways of toxicity. Such comparisons will determine if the “green” synthesis route genuinely mitigates adverse effects or merely shifts the toxicological profile.

Furthermore, the scope of future research should be expanded beyond aquatic systems to include terrestrial ecosystems, providing a more holistic understanding of the environmental fate and impact of green-synthesized nanomaterials across diverse biomes. Integrating life cycle assessments, from synthesis to disposal, and incorporating safe-by-design principles into the development of green nanotechnology will be paramount in ensuring a truly sustainable and responsible approach to the application of these innovative materials, minimizing potential ecological risks while maximizing their societal benefits. These efforts are crucial to bridge the gap between rapid nanotechnological advancement and the imperative for environmental protection.

## Figures and Tables

**Table 1:** Changes in the number and size of goblet cells in the epidermis in adult zebrafish following exposure to AgNPs. Values are mean ± SD of three replicates. Values with the letters within the same row are significantly different.

Item Group/Hour	Number of Goblet Cells/Hour			Size of Goblet Cells/(µm) Hour		
	24 h	48 h	96 h	24 h	48 h	96 h
Control 0	11.17 ± 3.148	12.67 ± 2.275	12.56 ± 2.617	12.71 ± 2.084	14.13 ± 2.453	13.9 ± 1.99
0.031 μg/L	20.00 ± 2.275 ^a^	13.17 ± 2.065	10.83 ± 1.339 ^a^	7.07 ± 1.22 ^a^	15.85 ± 2.369 ^a^	15.25 ± 1.609
0.250 μg/L	18.94 ± 2.287 ^a^	18.89 ± 2.298 ^a^	11.28 ± 1.074	13.13 ± 5.079	16.15 ± 2.298	13.73 ± 1.516
5.000 μg/L	12.00 ± 2.086	9.556 ± 1.423 ^a^	14.67 ± 2.635	11.57 ± 4.236	8.256 ± 1.382 ^a^	6.403 ± 1.583 ^a^

**Table 2:** Histopathological changes in fish exposed to AgNPs for 96 h were evaluated using arbitrary scores based on their severity (− none, + mild, ++ moderate, +++ severe).

Organ	Pathological Lesion Types Observed	0 μg/L	0.031 μg/L			0.250 μg/L			5.000 μg/L		
		−	24 h	48 h	96 h	24 h	48 h	96 h	24 h	48 h	96 h
Epidermal Tissues	Irregular structure of alarm cells	−	+	+	+	++	++	++	+++	+++	+++
	Shrunk cytoplasm alarm cells	−	+	+	+	++	++	++	+++	+++	+++
	PAS reaction	−	+++	+++	+++	++	++	++	+++	+++	+++
	AB (pH 2.5)	−	+++	+++	+++	+++	++	++	++	++	++

## Data Availability

Additional data supporting the manuscript are available from the corresponding author upon request.
